# Child and adolescent exposure to unhealthy food marketing across digital platforms in Canada

**DOI:** 10.1186/s12889-024-19094-5

**Published:** 2024-06-29

**Authors:** Monique Potvin Kent, Mariangela Bagnato, Lauren Remedios, Julia Soares Guimarães, Grace Gillis, Carolina Soto, Farah Hatoum, Meghan Pritchard

**Affiliations:** 1 School of Epidemiology and Public Health, Faculty of Medicine, University of Ottawa, 600 Peter Morand, Room 301J, Ottawa, ON K1G 5Z3 Canada; 2 Interdisciplinary School of Health Sciences, University of Ottawa, Ottawa, ON Canada

**Keywords:** Digital media, Unhealthy food marketing to youth, Food environment, Children, Adolescents

## Abstract

**Background:**

Children and adolescents are exposed to a high volume of unhealthy food marketing across digital media. No previous Canadian data has estimated child exposure to food marketing across digital media platforms. This study aimed to compare the frequency, healthfulness and power of food marketing viewed by children and adolescents across all digital platforms in Canada.

**Methods:**

For this cross-sectional study, a quota sample of 100 youth aged 6–17 years old (50 children, 50 adolescents distributed equally by sex) were recruited online and in-person in Canada in 2022. Each participant completed the WHO screen capture protocol where they were recorded using their smartphone or tablet for 30-min in an online Zoom session. Research assistants identified all instances of food marketing in the captured video footage. A content analysis of each marketing instance was then completed to examine the use of marketing techniques. Nutritional data were collected on each product viewed and healthfulness was determined using Health Canada’s 2018 Nutrient Profile Model. Estimated daily and yearly exposure to food marketing was calculated using self-reported device usage data.

**Results:**

51% of youth were exposed to food marketing. On average, we estimated that children are exposed to 1.96 marketing instances/child/30-min (4067 marketing instances/child/year) and adolescents are exposed to 2.56 marketing instances/adolescent/30-min (8301 marketing instances/adolescent/year). Both children and adolescents were most exposed on social media platforms (83%), followed by mobile games (13%). Both age groups were most exposed to fast food (22% of marketing instances) compared to other food categories. Nearly 90% of all marketing instances were considered less healthy according to Health Canada’s proposed 2018 Nutrient Profile Model, and youth-appealing marketing techniques such as graphic effects and music were used frequently.

**Conclusions:**

Using the WHO screen capture protocol, we were able to determine that child and adolescent exposure to the marketing of unhealthy foods across digital media platforms is likely high. Government regulation to protect these vulnerable populations from the negative effects of this marketing is warranted.

**Supplementary Information:**

The online version contains supplementary material available at 10.1186/s12889-024-19094-5.

## Introduction

The marketing of unhealthy foods high in saturated fat, sugar and sodium to children and adolescents has been identified as a determinant of food intake, food preferences, food requests, overweight and obesity [1–3]. Children and adolescents are heavily exposed to this marketing in a variety of media such as television and digital media, and in settings such as schools and recreation centres [4–7].

Digital media represents a growing source of youth’s exposure to unhealthy food/beverage (hereinafter referred to as food) marketing, as children and adolescents are heavy users of digital devices such as smartphones, tablets and laptops, spending 4 + hours on these devices daily [8]. Device access among children and adolescents is also ubiquitous. In 2021, 94% of 8–18 year old’s had access to a smartphone, 74% had access to a tablet and 87% had access to a laptop or desktop computer [8].

Unlike traditional forms of advertising, such as television and outdoor advertising, marketing in digital media is limitless; its reach spreads locally, globally and across multiple platforms [9]. The scope of marketing techniques in digital media is also broad, frequently blurring the lines between entertainment and promotion [10]. For instance, popular tactics such as social media influencer marketing, an emerging and highly effective digital marketing technique in which companies pay social media celebrities to promote their products, has been shown to increase children’s consumption and positive attitudes towards promoted products [11, 12]. Digital marketing is also adaptive due to user data tracking of sociodemographic information and online behaviours facilitated through artificial intelligence ensuring that marketing messages in this media are impactful and effective [13, 14]. Finally, digital marketing is interactive and the cost is low compared to traditional advertising on television, which entices food companies to shift their marketing budgets towards digital media [15–18].

Although an important media to monitor, only three studies internationally have estimated youth’s actual food marketing exposure on digital devices using screen capture methodologies. This methodology, where participant’s screens are recorded as they use them, is considered the gold standard for measuring exposure to digital marketing [19]. In Canada, data from 2019 examining child exposure to food marketing during 10 min of social media use on Facebook, Instagram, Snapchat, Twitter and YouTube estimated that children and adolescents view upwards of 1500 and 9000 instances of food marketing per year, respectively [20]. A recent Australian study examining food marketing on mobile devices using screen recordings estimated that youth aged 13–17 years view 8736 promotions per year, and a similar study investigating Mexican youth aged 6–19 years using 45-min screen recordings estimated children were exposed to 2461 promotions per year [21, 22]. In each of these studies, the majority of the foods viewed by children (> 90%) were classified as unhealthy [21, 22].

In 2018, the WHO European Office for the Prevention and Control of Noncommunicable Diseases developed the CLICK framework to help countries monitor digital food marketing to children [19]. Part of this framework includes a module, developed by our team, to estimate children’s exposure to unhealthy digital food marketing while they are online using a real-time screen capture methodology [19]. Such monitoring of both children and adolescents is necessary to inform stronger restrictions on food marketing to youth internationally particularly since adolescents are frequently excluded from such regulations [23]. In Canada, most of the country is self-regulated by the food industry through *the Food and Beverage Advertising Code (2023)* and the former *Canadian Children’s Food and Beverage Advertising Initiative (2007–2023)* [24, 25]. These codes seek to restrict unhealthy food marketing to children under 12 and 13, respectively, and though both initiatives extend to digital media, they both exclude social media and do not confer any protection to adolescents [24, 25]. This study aimed to estimate and compare the frequency, healthfulness and power of food marketing viewed by children and adolescents across all digital platforms in Canada using the recently developed WHO-Europe screen capture protocol. It was hypothesized that adolescents would be more frequently exposed to unhealthy food marketing across digital platforms compared to children, and most of the marketing would promote unhealthy products.

## Methods

### Study protocol

We conducted an observational study with 100 Canadian children (6–11 years) and adolescents (12–17 years) whose mother tongue was English or French (Canada’s official languages). Here it was important to include both children and adolescents as each group is vulnerable to unhealthy digital food marketing, spend different amounts of time on digital media and may choose different online activities and platforms [26–30]. Data on each age group are also required by governments to inform policy decisions as industry self-regulation and government regulations in this area have often focused on children under 12 or 13 years while excluding protections for adolescents [23]. Equal numbers of children and adolescents, and males and females were recruited both online and in-person by several methods including social media (i.e. paid Facebook ads, community organization social media, Twitter, Reddit and online snowball sampling), in-person at a local shopping mall, and using the market research company Leger [31]. Individuals were eligible to participate if they were a Canadian resident/citizen, 6–17 years old, able to read and understand English or French, use a smartphone or tablet during their leisure time, able to bring their smartphone or tablet to the study session, and have access to a desktop computer/laptop with a camera. Each participant attended one Zoom meeting with a researcher where they were asked to use their smartphone or tablet for 30-min, while sharing their device’s screen and being screen and device audio recorded. Participants were instructed to use their device as they normally would, but to avoid any e-mail, texting or banking applications to prevent sharing sensitive information. If any sensitive information was captured in the recording, participants were informed that they could ask for it to be deleted from their recording. Children aged 6–11 completed an online questionnaire with their parent and adolescents aged 12–17 completed this questionnaire independently. The questionnaire, which was adapted from previous research [20], queried sociodemographic information (i.e. age, sex, ethnicity, language, perceived income adequacy) and information on digital device ownership and usage habits on each type of digital device including smartphones, tablets, as well as laptops and desktop computers (e.g., “How long do you usually spend using a smartphone when you’re not at school or doing homework …on a typical weekday? (hr:min) …on a typical weekend day? (hr:min)) [20]. Informed parental consent and child assent were obtained, and participants were compensated with a $20 gift card. Ethics approval was obtained from a University of Ottawa research ethics board (H-11–21-7166).

### Identification and content analysis of food marketing instances

The screen capture recordings were reviewed by four research assistants (RAs) to collect all instances of food marketing. An instance of food marketing referred to any occasion where a branded food product or brand was mentioned or shown on the screen capture and was only recorded once per post if it appeared multiple times. It included brand/company marketing, branded product marketing and product representations (e.g., branded gummy bear shaped backpack). Each instance of food marketing was categorized according to type: brand marketing (no product featured) or product marketing; marketing platform (i.e. websites, mobile games, social media platform [e.g., Instagram, TikTok, or drawing apps]) and; food product category based on a modified version of the 2015 WHO-Europe Nutrient Profiling Model (Supplementary Table 1) [32]. In instances where a marketing instance contained multiple products, each featured product category was categorized.

### Identification of marketing techniques

A content analysis of marketing techniques, such as the presence of graphic effects, songs/music and use of spokes characters was conducted by trained RAs to assess the power of the marketing instances. Marketing power refers to the creative design and content of the marketing instance. Marketing instances were randomly distributed amongst four RAs and marketing techniques were coded as present or absent and only counted once per marketing instance (see definitions in Supplementary Table 1 adapted from previous research [20, 33]). Interrater reliability was established before coding began: each RA individually coded a 10% sub-sample of marketing instances from the database, establishing an inter-rater reliability of 0.91. Coding inconsistencies were resolved by consensus.

### Nutritional analysis

The nutritional information for each of the promoted products was collected through the following sources, in order of priority: 1) the company’s Canadian website; 2) Canadian food retailer websites (i.e. Loblaws Canada); 3) the company’s American website, or; 4) American food retailer websites (i.e. Walmart USA). For products where no flavour was specified, nutritional information was collected for the “original” or most plain version (e.g., “Häagen Dazs ice cream” – collected information for Häagen Dazs vanilla ice cream).

The healthfulness of promoted products and brands was classified using Health Canada’s proposed Nutrient Profile Model (NPM) from 2018, which classified foods and beverages as “of concern from an advertising perspective” (i.e. less healthy) or “not of concern from an advertising perspective” (i.e. healthier) [34]. This NPM classifies products according to their content of saturated fat, sodium and free sugars based on defined “low-in” thresholds for these nutrients (Supplementary Table 2). A product is considered less healthy if the content of at least one of these nutrients surpasses the “low-in” threshold and is considered healthier if none of these nutrients surpass the defined thresholds. In cases where a marketing instance contained multiple products, the advertisement was considered less healthy if at least one of the products in the marketing instance was classified as such. Brands were classified according to Health Canada’s “Food Brand/Restaurant Advertisement Decision list” (unpublished) which classifies brands based on the percentage of the brand’s products that would be subject to advertising restrictions in Canada. Brands are considered less healthy if > 50% of the brand’s products would be subject to advertising restrictions and healthier if < /= 50% of the brand’s products would be subject to advertising restrictions. Unclassified brands were excluded from the nutritional analysis (*n* = 29).

### Statistical analysis

Descriptive statistics were calculated using SPSS v.29.0.1.0 (IBM, 2023). Average time spent using a smartphone/tablet was calculated per child (6–11 years), per adolescent (12–17 years) and per youth (6–17 years) on weekdays and weekend days using daily time spent on these devices reported by participants. Time spent on laptops/desktops was excluded from this calculation since we measured exposure on smartphones or tablets only and it was judged that marketing exposure on laptops/desktops may vary given that different online activities (i.e. non-social media related activities) are typically undertaken on these devices. The following formulas were used to estimate the average daily, weekly and yearly exposure to food marketing per child, adolescent and youth:

Estimated average daily (weekday or weekend day) exposure to food marketing = (n marketing instances viewed by sample/n participants in sample)*(average mins spent using smartphone or tablet per day/30 min).

Estimated average weekly exposure to food marketing = [(estimated average exposure to food marketing per weekday*5 weekdays) + (estimated average exposure to food marketing per weekend day*2 weekend days)].

## Results

### Participant characteristics

A total of 100 participants (50 children, 50 adolescents) completed the study. As noted in Table 1, 51% were male, about half (54%) of participants self-identified as a majority ethnicity and 92% of participants were from middle-high income households. On average, adolescents spent the most time (hr:min) using smartphones and tablets during leisure time on a typical weekday (3:59) and a typical weekend day (5:35) compared to children (2:31 and 3:42, respectively).

**Table 1:** Sociodemographic characteristics of study sample (*n* = 100)

	**%**
**Sex**	
Male	51
Female	49
**Age Group**	
Children (6–11 years)	50
Adolescents (12–17 years)	50
**Ethnicity**	
Majority (White)	54
Minority^b^	46
**Perceived income adequacy**^**a**^	
Middle-high	92
Low	6
Don't know/missing	2
**Average time spent using a tablet or smartphone**	hr:min
Child weekday	2:31
Adolescent weekday	3:59
Child weekend	3:42
Adolescent weekend	5:35

^a^Middle-high includes ‘enough money’ and ‘more than enough money’. Low includes ‘not enough money’ and ‘barely enough money’

^b^Minority ethnicity includes the following groups: Indigenous, Black, East/Southeast Asian, South Asian, Latino, and Middle Eastern

### Youth’s overall marketing exposure

Overall, 226 instances of food marketing were viewed by 51% of youth (6–17 years old) in the sample (range of 1–46 marketing instances per participant), with children exposed to approximately 1.96 food marketing instances within a 30-min timeframe, while adolescents encountered an estimated 2.56 instances during the same period, on average (Table 2; Supplementary File 1). Overall, most (83%) marketing instances were viewed on social media platforms, particularly on YouTube (29%), Pinterest (23%; all instances from 2 participants) and TikTok (20%). Amongst children, 94% of marketing instances were viewed on social media, with Pinterest (37%), YouTube (35%) and TikTok (17%) dominating exposure. Amongst adolescents, 75% of marketing instances were viewed on social media, with the highest exposure being on YouTube (25%), followed by TikTok (22%) and Pinterest (13%).

**Table 2:** Frequency of food and beverage marketing instances viewed by children (6–11 years) and adolescents (12–17 years) in Canada in 30-min, by digital platform

Platform	Age Group		Total n(%)
	**Children (6-11yrs)** **n(%)**	**Adolescent (12-17yrs)** **n(%)**	
**Websites**	**0(0)**	**2(2)**	**2(1)**
**Mobile games**	**5(5)**	**24(19)**	**29(13)**
**Social media**	**92(94)**	**96(75)**	**188(83)**
Facebook	5(5)	0(0)	5(2)
Instagram	0(0)	14(11)	14(6)
Pinterest	36(37)	17(13)	53(23)
Snapchat	0(0)	4(3)	4(2)
TikTok	17(17)	28(22)	45(20)
Twitter	0(0)	1(1)	1(0)
YouTube	34(35)	32(25)	66(29)
**Drawing apps**	**1(1)**	**6(5)**	**7(3)**
**Total**	**98(43)**	**128(57)**	**226(100)**

Based on the reported time spent using smartphones and tablets amongst youth (Table 1) and the total frequency of food marketing exposures (Table 2), it was estimated that youth overall are exposed to an estimated 6023 marketing instances/youth/year, on average. By age group, children were exposed to an estimated 4067 marketing instances/child/year and adolescents were exposed to an estimated 8301 marketing instances/adolescent/year, on average (Supplementary File 1).

### Youth’s exposure to companies/brands featured in marketing instances

The most frequently featured companies/brands in food marketing instances viewed by youth overall were Mondelez (brand examples from sample: Oreo, Crispers & Cadbury) (8%), PepsiCo (e.g., Pepsi, Lay’s & Doritos) (8%), and Coca-Cola (e.g., Coca-Cola, Fairlife & Powerade) (7%) (Supplementary Table 3). For children, the top companies featured were Mondelez (12%), Coca-Cola (11%), and PepsiCo (5%). Among adolescents, PepsiCo (9%), Conagra Brands (e.g., BIGS) (5%), and Kellogg’s (e.g., Special K) (5%) were the most frequently featured companies in marketing instances.

### Healthfulness of youth’s exposure to food marketing

For both children and adolescents, the food categories that represented the highest levels of exposure included fast food restaurants (22%), savoury snacks (11%), chocolate/candy (11%), regular soft drinks (9%) and food delivery services/grocery stores (8%), while other food categories such as fruits/vegetables were rarely observed (Table 3). According to Health Canada’s NPM, 89% of both brands and products to which youth were exposed were classified as less healthy. In terms of age group, 87% and 90% of products/brands marketed to children and adolescents, respectively, were classified as less healthy (Table 4).

**Table 3:** Frequency of food and beverage products viewed by children (6–11 years) and adolescents (12–17 years) through marketing instances in Canada in 30-min, by food category

Food Category	Age Group		Total n(%)
	**Children** **(6-11yrs)** **n(%)**	**Adolescents** **(12-17yrs)** **n(%)**	
Fast food restaurants	25(22)	29(21)	**54(22)**
Chocolate and candy	12(11)	14(10)	**26(11)**
Savoury snacks	8(7)	18(13)	**26(11)**
Regular soft drinks	10(9)	13(10)	**23(9)**
Food delivery services/grocery stores	7(6)	12(9)	**19(8)**
Breakfast cereals	5(5)	8(6)	**13(5)**
Condiments	4(4)	9(7)	**13(5)**
Other beverages	5(5)	7(5)	**12(5)**
Cakes, cookies, and pastries	6(5)	4(3)	**10(4)**
Cheese	3(3)	3(2)	**6(2)**
Edible ices (ice cream, frozen yogurt, etc.)	2(2)	4(3)	**6(2)**
Energy drinks	2(2)	3(2)	**5(2)**
Processed meat/fish	5(5)	0(0)	**5(2)**
Entrees and ready-to-eat meals	3(3)	1(1)	**4(2)**
Non fast food restaurants	1(1)	3(2)	**4(2)**
Other (e.g., seasonings)	4(4)	0(0)	**4(2)**
Milk drinks	3(3)	0(0)	**3(1)**
Water	1(1)	2(2)	**3(1)**
Yogurts	1(1)	2(2)	**3(1)**
Butter/oils	2(2)	0(0)	**2(1)**
Pasta/grains	1(1)	1(1)	**2(1)**
Processed fruit/vegetables	1(1)	1(1)	**2(1)**
Breads	0(0)	1(1)	**1(0)**
Diet soft drinks	1(1)	0(0)	**1(0)**
Fresh and frozen meat/fish	0(0)	1(1)	**1(0)**
Frozen fruit/vegetables	0(0)	0(0)	**0(0)**
100% fruit juices	0(0)	0(0)	**0(0)**
**Total**	**112(45)**	**136(55)**	**248(100)**

^a^Total does not add to 226 because 16 marketing instances had multiple products/product categories

**Table 4:** Frequency of food and beverage marketing instances viewed by children (6–11 years) and adolescents (12–17 years) in Canada in 30-min, by Health Canada Nutrient Profile Model classification

	**Age group**						**Total**		
	**Children (6-11yrs)**			**Adolescents (12-17yrs)**					
	**Brands**	**Products**	**Both**	**Brands**	**Products**	**Both**	**Brands**	**Products**	**Both**
	**n(%)**	**n(%)**	**n(%)**	**n(%)**	**n(%)**	**n(%)**	**n(%)**	**n(%)**	**n(%)**
Healthier	4(21)	7(10)	11(13)	2(6)	9(12)	11(10)	6(12)	16(11)	22(11)
Less healthy	15(79)	61(90)	76(87)	31(94)	68(88)	99(90)	46(88)	129(89)	175(89)
**Total**	**19(100)**	**68(100)**	**87(100)**	**33(100)**	**77(100)**	**110(100)**	**52(100)**	**145(100)**	**197(100)**

^a^*N* = 29 brands could not be classified according to the HC M2K protocol and were considered missing

### Youth’s exposure to marketing techniques

For youth overall, the most frequently viewed marketing techniques were appealing graphic effects (20%), songs/music (13%), calls to action (11%), appeals to fun/cool (8%), and appeals to health/nutrition (6%) (Table 5). Among children specifically, the most frequently viewed marketing technique was appealing graphic effects (21%), followed by songs/music (14%), calls to action (8%), appeals to fun/cool (7%), and appeals to health/nutrition (7%). Among adolescents, the most frequently viewed marketing technique was appealing graphic effects (19%), followed by calls to action (13%), songs/music (12%), and appeals to fun/cool (8%).

**Table 5:** Frequency of marketing techniques featured in food and beverage marketing instances viewed by children (6–11 years) and adolescents (12–17 years) in Canada in 30-min

Marketing Technique	Age Group		Total n(%)
	**Children** **(6-11yrs)** **n(%)**	**Adolescents (12-17yrs)** **n(%)**	
Appealing graphic effects	40(21)	46(19)	**86(20)**
Songs/music	26(14)	29(12)	**55(13)**
Calls to action	15(8)	31(13)	**46(11)**
Appeals to fun/cool	13(7)	20(8)	**33(8)**
Appeals to health/nutrition	13(7)	13(5)	**26(6)**
Animations	8(4)	14(6)	**22(5)**
Price promotions	7(4)	14(6)	**21(5)**
Viral marketing	6(3)	14(6)	**20(5)**
Teen themes	5(3)	11(4)	**16(4)**
Use of celebrities	2(1)	14(6)	**16(4)**
Use of spokes/branded characters	10(5)	4(2)	**14(3)**
Presence of teens	6(3)	6(2)	**12(3)**
Appeals to convenience	6(3)	5(2)	**11(3)**
Child themes	5(3)	2(1)	**7(2)**
Cross-promotions	1(1)	6(2)	**7(2)**
Adult–child situations	5(3)	1(0)	**6(1)**
Presence of children	4(2)	2(1)	**6(1)**
Incentives/giveaways	3(2)	2(1)	**5(1)**
Appeals to energy	3(2)	1(0)	**4(1)**
Adult-teen situations	2(1)	1(0)	**3(1)**
Appeals to social enhancement	2(1)	1(0)	**3(1)**
Corporate social responsibility	3(2)	0(0)	**3(1)**
Presence or mention of gender	0(0)	3(1)	**3(1)**
Use of child or teen language	0(0)	3(1)	**3(1)**
Use of licensed characters	1(1)	2(1)	**3(1)**
Use of other cartoon characters	2(1)	1(0)	**3(1)**
Games	1(1)	1(0)	**2(0)**
Appeals to achievement	1(1)	0(0)	**1(0)**
Appeals to beauty	1(1)	0(0)	**1(0)**
Appeals to sex	0(0)	0(0)	**0(0)**
**Total**	**191(44)**	**247(56)**	**438(100)**

## Discussion

### Youth’s estimated exposure and overall marketing content

Interestingly, there was great variability in exposure rates within our sample of participants. While 51% were exposed to food marketing, 49% were not exposed at all. One participant viewed an exorbitant total of 46 food marketing instances in just 30 min, which likely reflects behavioural targeting and the individualized experience of digital media. Other studies in Canada and Mexico measuring food marketing exposure on digital devices have reported exposure by 72% and 70% of participants, respectively [20, 22]. Our rates could be lower compared to the previous Canadian study, as we asked participants to use their digital device as they normally would, whereas the previous study asked participants to spend 5 min each on two of their favourite social media applications. Social media applications in particular have been shown to contain high rates of food marketing [10, 20–22].

Overall, for youth that were exposed to instances of food marketing, exposure was high, with the majority of promoted products/brands being unhealthy. On average, adolescents viewed more marketing instances across digital platforms, at rates of 8301 marketing instances/adolescent/year, compared to 4067 marketing instances/child/year amongst children. These differences may be attributable to time spent on digital devices; compared to children, adolescents spent more time using digital devices (3:59–5:35 [hr:min] vs. 2:31–3:42 for children). The exposure rate for adolescents is consistent with previous Canadian data investigating youth exposure to unhealthy food marketing on selected social media applications using a 10-min screen capture sample which estimated that adolescents view more than 9000 marketing instances on social media per year [20]. Our results are also consistent with an Australian study that used multiple screen capture samples and estimated that adolescents aged 13–17 are exposed to 8736 marketing instances/child/year [21]. In Mexico however, adolescents have been estimated to view fewer food marketing instances, 2350 marketing instances/adolescent/year, which may be due to differences in industry marketing practices in low-middle income countries [22].

Our estimated rate of exposure for children (4067 marketing instances/child/year) is higher than previously found in Canada in 2019 (approximately 1500 marketing instances/child/year) using screen capture methodologies which may indicate that the food industry is increasingly focusing its advertising dollars on targeting children in digital media in Canada [20].

Overall, youth were most exposed to ultra-processed foods and sugar-sweetened beverages. The food category with the highest exposure was fast food; a food category that is associated with poor diet and weight gain amongst youth, increasing their risk for negative health outcomes such as cardiovascular disease, cancer and diabetes [35–38].

Youth were also exposed to a plethora of marketing techniques, which often blur marketing and entertainment, with the most frequent being those that may appeal more to youth, including appealing graphic effects (20%), songs/music (13%), calls to action (e.g., “visit link in bio”) (11%), and animations (5%) [10, 20]. Appeals to health/nutrition (6%) were also present (e.g., a YouTuber drinking Diet Coke and saying it’s healthier than normal Coke). Considering most promoted products were classified as unhealthy, the prominence of health appeals is problematic, as it falsely leads consumers to believe that the product is beneficial to their health [39–41].

### Why are we concerned about digital media marketing and youth?

It is worrisome from a public health perspective that adolescents in our study were exposed to such high levels of food marketing. Adolescents possess unique vulnerabilities to unhealthy digital marketing; they have increasing independence from parents, purchasing power, are highly susceptible to marketing due to their stage of neurocognitive development and spend many hours daily using digital devices [8, 26–28]. We found that during leisure time alone, adolescents spent just over 5.5 hours on smartphones and tablets on a typical weekend day. This was consistent with previous Canadian data which reported adolescents aged 10–15 years spend 3 + hours per day of their leisure time on weekends using a digital device [28]. We are equally as concerned about children’s exposure to unhealthy food marketing on digital media. Children also possess their own unique vulnerabilities, including difficulties identifying instances of marketing [29]. Due to the unique features of digital marketing, such as its interactivity, use of games, quizzes and polls and social media influencers, marketing can easily be misinterpreted as entertainment, making it difficult for children to identify and think critically about the marketing viewed [30, 42, 43].

### Policy implications

Our results reinforce previous data highlighting the failure of the current self-regulatory food marketing environment [33, 44, 45]. Interestingly, we found that all of the top companies to which youth were exposed, with the exception of one company, were signed on to the former voluntary self-regulatory initiative, the *Canadian Children’s Food and Beverage Advertising Initiative*, which was active when this data was collected [24]. This is not surprising given that social media (a major source of food marketing) is excluded from this pledge. Another industry code that was implemented in Canada in July 2023, the *Food and Beverage Advertising Code*, also excludes social media [25]. Our results highlight that any regulations that are crafted to restrict food marketing to children need to include such digital media platforms, as children are frequent users despite official age restrictions [46]. In April 2023, Health Canada released proposed regulations that would restrict unhealthy food marketing targeted at children under age 13 on television and in digital media [47]. This includes advertising to children on social media, websites, mobile applications, e-mail, streaming services and online games. No timeline has been established for the implementation of these regulations, however, this is a step in the right direction.

Worldwide restrictions on digital food marketing to children are either insufficient or non-existent. Due to the complexities surrounding digital marketing, a multifaceted, compulsory and internationally coordinated approach is paramount to protect youth from unhealthy food marketing in this media [14, 48]. Such restrictions are possible as many products that are harmful to children have been restricted on social media, including cannabis, vaping, alcohol, tobacco, gambling and weight loss products [46, 49, 50]. Government regulation in this area, coupled with independent monitoring and enforcement mechanisms would ensure that youth and their health are protected.

### Strengths/limitations

To our knowledge, this is the first study to estimate and compare the frequency, healthfulness and power of food marketing viewed by children and adolescents across all digital media channels in Canada. 30-min device recordings were used, which is the longest exposure measure in this research area in a Canadian context. This study also sampled both child and adolescent participants and had a high representation of ethnic minorities. The participants used their own device, or a borrowed device they use often, as they normally would and were not limited to specific social media applications, as has been done previously.

The limitations of this study included that most participants were from middle-high income families, and specific geographical data were not collected, which may restrict generalizability. Generalizability is also restricted by the nature of digital media itself, due to differences in behavioural targeting between participants. Additionally, as the device usage data collected from the questionnaires was self-reported and estimated, this could have introduced measurement error in the exposure calculations. Furthermore, since participants were being observed and recorded while using their devices, their normal digital behaviours may have been altered. A 30-min recording may also not accurately represent their normal exposure, though results mirrored those found in longer, repeated samples in Australia [21]. Also, although having access to a desktop/laptop computer for the session was an inclusion criterion to facilitate troubleshooting and provide clear digital device instruction, it was not completely necessary and could be removed in future research to generalize inclusion. Additionally, exposure calculations only considered smartphone and tablet usage data and excluded laptop/desktop usage which may have led to an underestimation of food marketing exposure. Finally, the healthfulness comparison may have been slightly skewed as a small number of brands (29 marketing instances) were not classified by the Health Canada NPM and were excluded from the nutritional analysis.

## Conclusion

Overall, we found that both children and adolescents were exposed to high volumes of unhealthy food marketing across all digital platforms in Canada. It is paramount that government regulation on unhealthy food marketing to youth on digital media are implemented to protect youth from the deleterious effects of food marketing of products high in sugar, fat and salt. Continued monitoring of the dynamic digital food marketing landscape using the CLICK framework is recommended to measure changes in the frequency and nature of digital food marketing to youth over time and foster international comparisons.

### Supplementary Information

Supplementary Material 1: Supplementary Table 1. Marketing technique coding manual. Supplementary Table 2. Health Canada Nutrient Profile Model Thresholds. Supplementary Table 3. Top food and beverage companies featured in food and beverage marketing instances viewed by children (6-11yrs) and adolescents (12-17yrs) in Canada in 30-min. Supplementary File 1. Calculations for average time spent on digital devices and daily, weekly and yearly exposure rates per child/adolescent/youth.

## Data Availability

No datasets were generated or analysed during the current study.

## Abbreviations

WHO: World health organization
NPM: Nutrient profile model
RAs: Research assistants
Ads: Advertisements
Hr: Hours
Min: Minutes
