# Supplementary material for: Child and adolescent exposure to unhealthy food marketing across digital platforms in Canada
Source: BMC Public Health. 2024 Jun 29;24:1740. doi: 10.1186/s12889-024-19094-5 (PMC11218052; doi:10.1186/s12889-024-19094-5)
Supplement: Supplementary file 1 — Supplementary Material 1: Supplementary Table 1. Marketing technique coding manual. Supplementary Table 2. Health Canada Nutrient Profile Model Thresholds. Supplementary Table 3. Top food and beverage companies featured in food and beverage marketing instances viewed by children (6-11yrs) and adolescents (12-17yrs) in Canada in 30-min. Supplementary File 1. Calculations for average time spent on digital devices and daily, weekly and yearly exposure rates per child/adolescent/youth. [file 12889_2024_19094_MOESM1_ESM.docx]

**Supplementary Table 1. Marketing technique coding manual**

| \| **VARIABLE** \| **DESCRIPTION** \| **CODING** \| \| --- \| --- \| --- \| | | |
| --- | --- | --- | --- | --- | --- |
| Food product type | If food/bev or brand ad, identify type (modified from WHO-Europe NPM) | 1=Chocolate and candy  2=Cakes, cookies, and pastries  3 Savoury snacks  4=100% fruit juices  5=Milk drinks  6=Energy drinks  7=Regular soft drinks  8=Diet soft drinks  9=Water  10=Other beverages  11=Edible ices (ice cream, frozen yogurt, etc.)  12=Breakfast cereals  13=Yogurts  14=Cheese  15=Entrees and ready-to-eat meals  16=Butter/oils  17=Breads  18=Pasta/grains  19=Fresh and frozen meat/fish  20=Processed meat/fish  21=Frozen fruit/veg  22=Processed fruit/veg  23=Condiments  24=Fast food restaurants  25=Non-fast food restaurants  26=Alcohol  27=Food delivery services/grocery stores  28=Other – specify  29=Mixed – specify |
| Reference to or presence of children | Features child (12 yrs or younger)  Reference to child not physically present or actual presence of child in the marketing instance | 0=No  1=Yes |
| Reference to or presence of teens | Features a teen (13-17yrs)  Reference to teen not physically present or actual presence of teen in the marketing instance | 0=No  1=Yes |
| Reference to or presence of gender | Marketing instance refers to gender in either a statement “this product is great for young girls” or it refers to an item that displays a specific gender  E.g., girls jumping rope on chocolate milk carton | 0=No  1=Yes |
| Use of child or teen language | Marketing instance uses language that is associated with children, that is frequently used by children, or that is directed at children.  E.g., “OMG”, “dope”, etc. | 0=No  1=Yes |
| Adult-child situations | Marketing instance features situations that play on the parent-child relationship or other authority-based relationship (e.g., coach-child, teacher-child). | 0=No  1=Yes |
| Adult-teen situations | Marketing instance features situations that play on the parent-teen relationship or other authority-based relationship (e.g., coach-teen, teacher-teen). | 0=No  1=Yes |
| Child themes | Marketing instance uses themes that are commonly associated with children such as fantasy, magic, mystery, suspense, adventure, zoo animals, virtual worlds, etc. This could include references to, or the incorporation of, popular trends in children’s interests of preferences, which may vary year-to-year or based on geographic location. This needs to be a deliberate inclusion of child themes to market the product. If there is a child present in the post, then child themes are also always present. | 0=No  1=Yes |
| Teen themes | Marketing instance uses themes that are commonly associated with teens, such as themes linked to high school, social media, ‘hanging-out’, popularity, fashion, risk-taking, independence, etc. This could include references to, or the incorporation of, popular trends in teens interests or preferences, which may vary year-to-year or based on geographic location. This needs to be a deliberate inclusion of teen themes to market the product. If there is a teen present in the post, then teen themes are also always present. | 0=No  1=Yes |
| **APPEALS** | | |
| Appeals to fun/cool | Marketing instance makes appeals to the food or beverage item being fun or funny, having fun while eating the product, being happy, humour or coolness/novelty. This could include depictions of the food itself doing something fun, or depictions of the food in motion, for example, a cookie diving into milk, or candies ‘exploding’ out of ice cream, juggling food products, someone spinning Oreos on their finger, etc. It can also be part of the name such as “Fun Dip” or “Kool Kreatures”. | 0=No  1=Yes |
| Appeals to health/nutrition | Marketing instance makes explicit appeals related to the healthfulness or nutritional quality of the product, its ability to promote wellness, growth, strength, or physical activity. The post can also make implicit appeals to health or nutrition, such as the product being displayed alongside “healthy foods” (e.g., fresh fruit being depicted in an ad for breakfast cereal), or the product being shown consumed by children while participating in physical activity. E.g., the post includes health and nutrition claims/symbols, as well as claims or symbols referring to the product being organic or natural. | 0=No  1=Yes |
| Appeals to social enhancement | Marketing instance highlights the product’s ability to enhance making friends, peer acceptance, or being social with others.  E.g., Coca-Cola advertisements that feature people giving friends Coke’s with their names on them. | 0=No  1=Yes |
| Appeals to convenience | Marketing instance highlights that the product is easy to carry or bring with you, easy to prepare, easy to eat, easy to share, etc. | 0=No  1=Yes |
| Appeals to sex | The marketing instance includes aspects of romance, sex, or hypersexuality to market a product. E.g., Paris Hilton wearing a bikini while eating a Carl’s Jr Burger. | 0=No  1=Yes |
| Appeals to beauty | Marketing instance includes aspects of beauty or attractiveness to market the product. E.g., An influencer doing their makeup while consuming a drink or food product. | 0=No  1=Yes |
| Appeals to energy | Marketing instance references or utilizes energy as a selling point e.g., “Red Bull gives you wings” | 0=No  1=Yes |
| Appeals to achievement | Marketing instance highlights the product’s ability to help with achievement or accomplishment or indicates this product has helped with success successful. E.g., Mentos that show problem solving or achievement because of consuming the product; Grey Poupon showing status or achievement. | 0=No  1=Yes |
| **CHARACTERS** | | |
| Use of spokes characters | A fictional/cartoon character that is defined by a set of human attributes and characteristics to give the brand a unique personality. E.g., Tony the Tiger, Pillsbury Doughboy, etc. | 0=No  1=Yes |
| Use of licensed characters | A licensed character involves licensing the rights from the owner of the cartoon character to place images on a product. E.g., using Spiderman on a Luncheables package. | 0=No  1=Yes |
| Use of other cartoon characters | Cartoons other than spokes or licensed characters are depicted in marketing instance (e.g., cartoon characters featured in Red Bull commercial) | 0=No  1=Yes |
| Use of celebrities | Presence or reference to a celebrity (e.g., actor, athlete, musician, influencer, etc.) | 0=No  1=Athletes  2=Actors  3=Musicians  4=Other (e.g., influencer) |
| **PROMOTIONS** | | |
| Cross-promotions | Marketing instance features cross-promotions to movies/sporting events/TV shows etc. other than one of the types of characters or celebrities described above. E.g., the marketing instance features aspects of a well-known fictional world, without specifically including the fictional characters.  Note: these may appear in addition to the presence of any characters described above.  Does NOT include promotion of other food or non-food brands (e.g., Uber Eats) or to other food products. | 0=No  1=Yes |
| Price promotions | Marketing instance includes a price-promotion or premium, including discounted prices on other merchandise included with the purchase of a food or beverage product. E.g., Charlie D’Amelio offers a discount on makeup if you buy Takis. This also includes if the price of a product is mentioned in a post (e.g., Cheetos cost $10!) | 0=No  1=Yes |
| Incentives/giveaways | Marketing instance promotes contests, prizes, or giveaways available with or without purchase. E.g., Addison Rae will give away $1000 to the first 100 people that comment on her post. | 0=No  1=Yes |
| Calls-to-action | Marketing instance encourages consumers to buy the product, participate in a campaign, visit a product/brand/company website, social media, or games-based brand website. Also includes the promotion of opportunities to “join”, “become a member”, complete a quiz, poll, or survey. E.g., Charli D’Amelio will encourage you to check out her Linktree and sign up for an account.   E.g., Other examples:  1. The caption of a food ad on social media includes “link in bio”.  2. The caption of a food commercial for M&Ms on YouTube includes “learn more about the purple M&M at mms.com”  3. A pop-up ad for Special K cereal includes a swipe up link and the caption “visit site” | 0=No  1=Yes |
| Corporate social responsibility | Marketing instance makes appeals to sustainability, philanthropy or contributing to other social causes. E.g., For every like on this post I (the influencer) will donate a dollar to cleaning up beaches. | 0=No  1=Yes |
| Viral marketing | Prompts viewers to engage with the brand by commenting, replying, sharing information with their peers (peer-to-peer marketing), re-posting content to their own feeds, tagging friends, or using specific hashtags. E.g., “Be sure to share this post with all of your friends”.    A hashtag of a company is displayed either in the description, comments section, or on the screen.  E.g., #wendys #invisalign.  Includes tagging a brand or other influencer (>5k followers). | 0=No  1=Yes |
| Games | Presence of games or activities within the post (including on packaging, marketing display, etc.). E.g., MrBeast is playing a video game while drinking Coke. | 0=No  1=Yes |
| **EFFECTS** | | |
| Songs/music | Music is used in the ad. E.g., songs, jingles, sound effects, etc. | 0=No  1=Yes |
| Animations | Animations are used in the marketing instance to make the product appealing. E.g., the use of cartoons to interact with the product. | 0=No  1=Yes |
| Appealing graphic effects | Graphic imagery is used in the post or to enhance the display of the product. E.g., bright colours, eye-catching backgrounds, fonts, etc. Visual effects are used in the post or to market the product. E.g., explosions, lights, fast cutting, slow motion, dynamic images, etc. | 0=No  1=Yes |

**Supplementary Table 2. Health Canada Nutrient Profile Model Thresholds**

|  | **Nutrient** | **Thresholds for foods** | **Thresholds for main dishes with a RA above 200g** |
| --- | --- | --- | --- |
| **Low in:** | Saturated Fat | A total of 2 g SFA per RA or serving of stated size, whichever is the greater and ≤ 15% energy from the SFA | A total of 2 g SFA per 100g and ≤ 15% energy is from the SFA |
|  | Sodium | 140 mg per RA or serving of stated size whichever is the greater or 140 mg per 50 g of the product if the RA is ≤ 30g or 30 mL | 140 mg per 100g |
|  | Sugars | 5 g per RA or serving of stated size whichever is the greater or 5 g per 50 g of the product if the RA is ≤ 30g or 30 mL | 5 g per 100g |
| ^a^Source: Health Canada. Health Canada's Proposed Nutrient Profile Model for Restricting Marketing to Children. 2019. Unpublished [cited 2023 June 7]. | | | |

**Supplementary Table 3. Top food and beverage companies featured in food and beverage marketing instances viewed by children (6-11yrs) and adolescents (12-17yrs) in Canada in 30-minutes**

|  | **Age Group** | |  |
| --- | --- | --- | --- |
| **Company** | **Children**  **(6-11yrs)**  **n(%)** | **Adolescents**  **(12-17yrs)**  **n(%)** | **Total**  **n(%)** |
| 7-Eleven | 1(1) | 0(0) | **1(0)** |
| A&W | 3(3) | 2(1) | **5(2)** |
| Alchemist Restaurant | 0(0) | 1(1) | **1(0)** |
| B&G Foods | 1(1) | 0(0) | **1(0)** |
| Bai | 1(1) | 0(0) | **1(0)** |
| Barcel | 0(0) | 4(4) | **4(2)** |
| Bel Groupe | 1(1) | 2(1) | **3(1)** |
| Boston Pizza | 0(0) | 1(1) | **1(0)** |
| Burger Factory Canada | 1(1) | 0(0) | **1(0)** |
| Candy Dynamics Inc. | 0(0) | 1(1) | **1(0)** |
| Carl's Jr. | 0(0) | 1(1) | **1(0)** |
| Charlie Brown's Fresh Grill Steakhouse | 1(1) | 0(0) | **1(0)** |
| Chef's Plate | 0(0) | 1(1) | **1(0)** |
| Chick-fil-A | 0(0) | 1(1) | **1(0)** |
| CLIF | 1(1) | 0(0) | **1(0)** |
| Coca-Cola | 12(11) | 6(4) | **18(7)** |
| Conagra Brands | 0(0) | 7(5) | **7(3)** |
| Cook It | 0(0) | 2(1) | **2(1)** |
| Cybrpunk | 0(0) | 1(1) | **1(0)** |
| Dairy Queen | 1(1) | 3(2) | **4(2)** |
| Domino's | 0(0) | 2(1) | **2(1)** |
| DoorDash | 0(0) | 6(4) | **6(2)** |
| Durkee-Mower | 1(1) | 0(0) | **1(0)** |
| Edo Japan | 1(1) | 0(0) | **1(0)** |
| Fatburger | 0(0) | 5(4) | **5(2)** |
| Ferrero SpA | 1(1) | 1(1) | **2(1)** |
| Fiji | 0(0) | 1(1) | **1(0)** |
| Flowers Foods Inc. | 0(0) | 1(1) | **1(0)** |
| General Mills | 4(4) | 5(4) | **9(4)** |
| Genshin Impact Global | 1(1) | 0(0) | **1(0)** |
| Happy Planet Foods | 0(0) | 1(1) | **1(0)** |
| Hard Stones Grill | 1(1) | 0(0) | **1(0)** |
| Haribo | 1(1) | 0(0) | **1(0)** |
| Hood Mart Restaurant | 0(0) | 1(1) | **1(0)** |
| Impact Confections | 0(0) | 1(1) | **1(0)** |
| Kellogg's | 5(5) | 7(5) | **12(5)** |
| Kerrygold | 1(1) | 0(0) | **1(0)** |
| Keurig Dr. Pepper | 1(1) | 2(1) | **3(1)** |
| KFC | 0(0) | 1(1) | **1(0)** |
| Kraft Heinz | 4(4) | 2(1) | **6(2)** |
| Krinos Foods | 1(1) | 0(0) | **1(0)** |
| La Fourmi Bionique | 1(1) | 0(0) | **1(0)** |
| Lactalis Canada | 1(1) | 4(4) | **5(2)** |
| Lindt | 0(0) | 3(2) | **3(1)** |
| Little Caesars | 1(1) | 0(0) | **1(0)** |
| Loblaws Companies | 3(3) | 0(0) | **3(1)** |
| Maple Leaf Foods | 4(4) | 0(0) | **4(2)** |
| Mars Wrigley | 1(1) | 3(2) | **4(2)** |
| McCormick & Company | 4(4) | 6(4) | **10(4)** |
| McDonald's | 5(5) | 6(4) | **11(4)** |
| Mizkan | 0(0) | 1(1) | **1(0)** |
| Mondelez | 13(12) | 6(4) | **19(8)** |
| Monster Energy | 1(1) | 1(1) | **2(1)** |
| Moxies | 0(0) | 1(1) | **1(0)** |
| Mr. Pizza | 1(1) | 0(0) | **1(0)** |
| MrBeast Burger | 1(1) | 0(0) | **1(0)** |
| Natrel | 1(1) | 0(0) | **1(0)** |
| Nestle | 2(2) | 4(4) | **6(2)** |
| Ontario Dairy | 1(1) | 0(0) | **1(0)** |
| Open Nature | 0(0) | 1(1) | **1(0)** |
| PepsiCo | 6(5) | 13(9) | **19(8)** |
| Pizza Hut | 0(0) | 2(1) | **2(1)** |
| Post | 0(0) | 1(1) | **1(0)** |
| Prime Hydration | 0(0) | 1(1) | **1(0)** |
| Red Bull | 1(1) | 1(1) | **2(1)** |
| Snyder's-Lance | 1(1) | 0(0) | **1(0)** |
| Sobeys | 1(1) | 2(1) | **3(1)** |
| Starbucks | 3(3) | 3(2) | **6(2)** |
| Subway | 1(1) | 3(2) | **4(2)** |
| The Alley | 0(0) | 1(1) | **1(0)** |
| The Hershey Company | 3(3) | 3(2) | **6(2)** |
| The PUR Company Inc. | 0(0) | 1(1) | **1(0)** |
| Tim Hortons | 5(5) | 2(1) | **7(3)** |
| Voilà | 1(1) | 1(1) | **2(1)** |
| Walmart | 3(3) | 0(0) | **3(1)** |
| Wendy's | 2(2) | 2(1) | **4(2)** |
| **Total** | **112(100)** | **141(100)** | **253(100)** |

**Supplementary File 1. Calculations for average time spent on digital devices and daily, weekly and yearly exposure rates per child/adolescent/youth**

***Average time spend on digital devices:***

**Children (6-11 years):**

***Weekday:***

- Smartphone avg. = **82.1 mins**
- Tablet avg. = **68.6 mins**

**Overall device weekday avg. = 150.7 mins**

***Weekend day:***

- Smartphone avg. = **104.9 mins**
- Tablet avg. = **116.7 mins**

**Overall device weekend day avg. = 221.6 mins**

**Adolescents (12-17 years):**

***Weekday:***

- Smartphone avg. = **207.8 mins**
- Tablet avg. = **30.9 mins**

**Overall device weekday avg. = 238.7 mins**

***Weekend day:***

- Smartphone avg. = **284.1 mins**
- Tablet avg. = **50.8 mins**

**Overall device weekend day avg. = 334.9 mins**

**Overall youth (6-17 years):**

- Weekday avg. = (150.7 mins child + 238.7 mins adolescent)/2 = **194.7 mins**
- Weekend day avg. = (221.6 mins child + 334.9 mins adolescent)/2 = **278.3 mins**

***Daily, weekly and yearly marketing instance exposure rates:***

**Marketing instance exposure rate per child (6-11 years):**

***Per weekday:***

(98 marketing instances/50 children)*(150.7 mins/30 mins)

=1.96*5.02

**=9.8 marketing instances/child/weekday**

***Per weekend day:***

(98 marketing instances/50 children)*(221.6 mins/30 mins)

=1.96*7.39

**=14.5 marketing instances/child/weekend day**

***Per week overall:***[(9.8 marketing instances/child/weekday*5 weekdays)+(14.5 marketing instances/child/weekend day*2 weekend days)]

**=78 marketing instances/child/week**

***Per year overall:***

78 marketing instances/child/week*52.143 weeks

**=4067 marketing instances/child/year**

**Marketing instance exposure rate per adolescent (12-17 years):**

***Per weekday:***

(128 marketing instances/50 adolescents)*(238.7 mins/30 mins)

=2.56*7.96

**=20.4 marketing instances/adolescent/weekday**

***Per weekend day:***

(128 marketing instances/50 adolescents)*(334.9 mins/30 mins)

=2.56*11.16

**=28.6 marketing instances/adolescent/weekend day**

***Per week overall:***

[(20.4 marketing instances/adolescent/weekday*5 weekdays)+(28.6 marketing instances/adolescent/weekend day*2 weekend days)]

**=159.2 marketing instances/adolescent/week**

***Per year overall:***

159.2 marketing instances/adolescent/week*52.143 weeks

**=8301 marketing instances/adolescent/year**

**Marketing instance exposure rate per youth overall (6-17 years):**

***Per weekday:***

(226 marketing instances/100 youth)*(194.7 mins/30 mins)

=2.26*6.49

**=14.7 marketing instances/youth/weekday**

***Per weekend day:***

(226 marketing instances/100 youth)*(278.3 mins/30 mins)

=2.26*9.28

**=21.0 marketing instances/youth/weekend day**

***Per week overall:***

[(14.7 marketing instances/youth/weekday*5 weekdays)+(21.0 marketing instances/youth/weekend day*2 weekend days)]

**=115.5 marketing instances/youth/week**

***Per year overall:***

115.5 marketing instances/youth/week*52.143 weeks

**=6023 marketing instances/youth/year**
